# Small area analysis methods in an area of limited mapping: exploratory geospatial analysis of firearm injuries in Port-au-Prince, Haiti

**DOI:** 10.1186/s12942-023-00337-4

**Published:** 2023-08-18

**Authors:** Athanasios Burlotos, Tayana Jean Pierre, Walter Johnson, Seth Wiafe, PROTRA Haiti Group, Corinne Pean, Monet McCalla, Carl Stephane Lominy, Jean Antoni Sebastien Lefevre, Yvelie Saint Lot, Jean Wilguens Lartigue, Alence Therone, Carolina Torres, Gabrielle Cahill, Michelle Joseph

**Affiliations:** 1 Department of Emergency Medicine, Boston Medical Center, Boston, MA USA; 2 Harvard T.H. Chan School of Public Health, Boston, MA USA; 3 Universite Notre Dame D’Haiti, Faculte de Medecine, Port-au-Prince, Haïti; 4 Loma Linda University School of Public Health, Loma Linda, CA USA; 5 Clinical Trials Unit, University of Warwick, Coventry, CV2 2DX United Kingdom; 6 Present Address: Department of Global Health and Social Medicine, Harvard Medical School, Boston, MA USA; 7 Program in Global Surgery and Social Change, Harvard Medical School, Boston, MA USA

## Abstract

**Background:**

The city of Port-au-Prince, Haiti, is experiencing an epidemic of firearm injuries which has resulted in high burdens of morbidity and mortality. Despite this, little scientific literature exists on the topic. Geospatial research could inform stakeholders and aid in the response to the current firearm injury epidemic. However, traditional small-area geospatial methods are difficult to implement in Port-au-Prince, as the area has limited mapping penetration. Objectives of this study were to evaluate the feasibility of geospatial analysis in Port-au-Prince, to seek to understand specific limitations to geospatial research in this context, and to explore the geospatial epidemiology of firearm injuries in patients presenting to the largest public hospital in Port-au-Prince.

**Results:**

To overcome limited mapping penetration, multiple data sources were combined. Boundaries of informally developed neighborhoods were estimated from the crowd-sourced platform OpenStreetMap using Thiessen polygons. Population counts were obtained from previously published satellite-derived estimates and aggregated to the neighborhood level. Cases of firearm injuries presenting to the largest public hospital in Port-au-Prince from November 22nd, 2019, through December 31st, 2020, were geocoded and aggregated to the neighborhood level. Cluster analysis was performed using Global Moran’s I testing, local Moran’s I testing, and the SaTScan software. Results demonstrated significant geospatial autocorrelation in the risk of firearm injury within the city. Cluster analysis identified areas of the city with the highest burden of firearm injuries.

**Conclusions:**

By utilizing novel methodology in neighborhood estimation and combining multiple data sources, geospatial research was able to be conducted in Port-au-Prince. Geospatial clusters of firearm injuries were identified, and neighborhood level relative-risk estimates were obtained. While access to neighborhoods experiencing the largest burden of firearm injuries remains restricted, these geospatial methods could continue to inform stakeholder response to the growing burden of firearm injuries in Port-au-Prince.

## Background

Traumatic injuries are a leading cause of death and disability for young people worldwide, with low-income and middle-income countries facing the highest burden [1]. In Haiti, from 1990 to 2017, interpersonal violence was the 3rd highest contributor to Disability-adjusted Life Years (DALYs) in males aged 15–49, behind only road traffic injuries and HIV/AIDS [2]. In recent years, the rates of interpersonal violence have spiked considerably [3]. The health system in Port-au-Prince (PAP) faces a large burden of firearm injuries.

In this context, the current geospatial study was conducted as part of the pre-implementation phase of Project Trauma Haiti (PROTRA Haiti), a multistage effort to improve the quality of trauma care delivery and increase trauma care capacity in Haiti. PROTRA Haiti is a multi-institutional collaboration between established Haiti medical societies and Harvard Medical School, led by Harvard’s Program for Global Surgery and Social Change, Association Haitienne de Chirurgie, Comité de Trauma, and Société Haïtienne de Médecine d’Urgence et de Catastrophe. The first analysis of the pre-implementation phase, an epidemiological analysis of patients with traumatic injuries that had attended a large public hospital in Port-au-Prince, provided quantitative evidence on the large burden of firearm injuries overwhelming the capital [4]. Next steps in the project which may be informed by this geospatial analysis include educational interventions, as well as improvement of the record-keeping and data collection systems used in the emergency departments at partnering institutions.

In other contexts, it is known that firearm injuries have been shown to display epidemiologic behavior similar to infectious diseases. Patients who are victims of a non-self-inflicted firearm injury are at an increased risk of becoming perpetrators of gun violence [5]. Similarly, violence can often spread through human networks, leading to periods of increased violence analogous to outbreaks of an infectious disease [6]. Despite the large burden of disease, there is a paucity of research to characterize the epidemiology of firearm injuries in Haiti.

Given this context, the objectives for this study were the following: First, to evaluate the feasibility of geospatial analysis in Port-au-Prince, an area with limited mapping penetration. Secondly, this study seeks to understand the specific limitations faced in this context, which will be used to guide future data collection, implementation of interventions, and geospatial research in Port-au-Prince. Lastly, the study seeks to explore the geospatial epidemiology of firearm injuries in patients presenting to the largest public hospital in Port-au-Prince, specifically evaluating for clusters of firearm injuries.

## Methods

### Study design

The study consisted of geospatial analysis of patients with firearm injuries, including both exploratory spatial analysis and spatial cluster analysis. Geospatial analysis was performed using neighborhoods, which were estimated using data from the crowdsourced map platform OpenStreetMap [7]. This was done to improve the resolution of the geospatial analysis beyond the level of communal sections, which is the smallest administrative boundary in the city.

### Setting

This study took place in Port-au-Prince, the capital and most populous city in Haiti. The population of the city is estimated to be 987,311 people, with 2,618,894 people in the larger metropolitan area [8]. Haiti is classified as a middle-income country [9]. Despite this, due to its colonial history, frequent natural disasters, and political instability, Haiti has the lowest GDP of the Latin America and Caribbean region [10]. Within Haiti, levels of income inequality are some of the highest in the region, with most extreme poverty occurring in rural regions [10].

### Data sources and variables

Clinical data, crowdsourced maps, and satellite-derived population estimates constituted the data sources which were combined for this study to facilitate conducting research in a setting with a low penetration of mapping. Information for firearm injury patients and non-firearm injury patients was obtained from the emergency department logbooks from Hôpital de l’Université d’Etat d’Haïti (HUEH), the largest public hospital in Haiti. Scanned handwritten HUEH logbooks were transcribed by the PROTRA Haiti Group. Data were quality checked (< 1% error rate), cleaned, grouped, and joined using the R Statistical Software [11] through an iterative process. Variables extracted for use in geospatial analysis included the emergency department visit date (giving the resulting dataset a temporal resolution of 1 day), patient diagnosis (coded as firearm injury or not a firearm injury), and a free text patient home address field. Prior research has demonstrated that home addresses can serve as a proxy for the location of injury for trauma patients [12].

The address field was geocoded using Google’s geocoding application programming interface (API), [13] which was executed via an R script utilizing the *ggmap* package, [14] and then reviewed by Dr. Jean Pierre, who has local expertise in the topography of the city through prior work in urban planning. Results from the geocoding API output were not filtered based on API output parameters, given the uncertainty of the APIs performance in Port-au-Prince. Additionally, filtration was unnecessary since each output entry from the API was reviewed manually by Dr. Jean Pierre to ensure accuracy. Manual review by Dr. Jean Pierre consisted of three steps. First, the free text fields containing address information were reviewed to ensure sufficient information was available for inclusion in the analysis at the neighborhood level. Given variable completeness of addresses in the free text field, addresses which could not be confidently located at the neighborhood level were excluded. Second, for addresses which were included, Dr. Jean Pierre then reviewed the matched GPS coordinates and compared this with the free text address. To facilitate this comparison, Dr. Jean Pierre referenced a large-scale map of the generated neighborhoods, as well as digital maps including Google Maps and OpenStreetMap. For data points which matched correctly, this completed the manual review. For complete addresses which matched incorrectly, there was an additional third step. For these locations, Dr. Jean Pierre manually appended a GPS coordinate for a point corresponding to the address. This was done by Dr. Jean Pierre manually placing a pin on the correct location using Google Maps, and then appending the GPS coordinate for the pin to the dataset. In these instances, the corrected, manually appended GPS coordinate was used for all data analysis. This process of manual review allowed the correction of GPS coordinates with enough precision to be accurately aggregated at the neighborhood level, without utilizing additional field work to geocode address fields. Furthermore, manual review and correction of GPS coordinates by Dr. Jean Pierre was needed to reduce systematic exclusion of patients from informal settlements, for which it was observed that the API performed poorly. Given safety concerns in many of the areas most impacted by firearm injuries, field coding of addresses using a GPS enabled device would not have been possible.

Data which were used to construct the neighborhood estimations was derived from OpenStreetMap, [7] and was determined to be the most accurate data source available for information on neighborhoods. In Port-au-Prince, neighborhoods were encoded in OpenStreetMap as a tag of the place parameter called “suburb”, defined as “a part of a town or city with a well-known name and often a distinct identity” [15]. Suburbs were encoded as nodes in OpenStreetMap. A list of suburbs in Haiti was downloaded from OpenStreetMap using the Overpass Query Service [16] as a *geojson* file. The query parameters were developed with the assistance of the R *osmdata* package, [17] and the resulting *geojson* file was imported using the R *sf* package [18]. As recent census data were not available, population estimates for Haiti derived from satellite imagery were obtained from WorldPop. Specifically, the 2020 constrained, top-down dataset was used [19]. The 2020 dataset was selected due to the best overlap with the study period. The constrained dataset was selected over the unconstrained as WorldPop reports that the unconstrained dataset tends to underestimate the population in urban areas. Lastly, the top-down dataset was chosen to facilitate comparison with other literature that may rely on United Nations reported population estimates. Furthermore, the use of remote sensing population data enabled the construction of neighborhoods that were smaller than available administrative boundaries in Haiti. The constrained population count format from WorldPop was obtained as a raster image stored as a *geotif* file with a resolution of 100 m. Population data in the raster format was summed and aggregated in vector format at the neighborhood level using the function *raster::extract* available in the R package *raster* [20]*.* Lastly, a shapefile for existing administrative boundaries in Haiti was downloaded from the Humanitarian Data Exchange [21].

### Inclusion criteria and bias

**Fig. 1:**
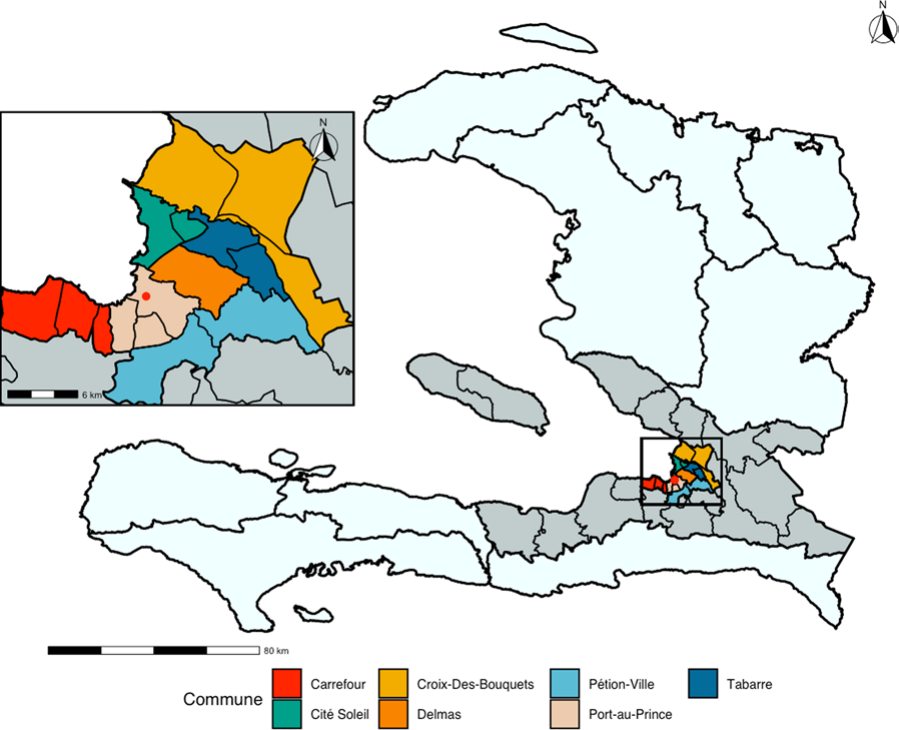
Definition of the study area and demonstration of the three administrative boundaries in Haiti. Boundaries within the study area reflect communal sections. The department of Ouest is shaded dark gray and shows boundaries between communes. The light blue area represents Haiti’s other departments, the highest administrative level. The red dot marks the location of HUEH. Graphic by author

All patients which presented to the emergency department of HUEH from November 22nd, 2019, through December 31st, 2020, were considered eligible for the study. Additional inclusion criteria for the geospatial analysis were diagnosis of firearm injury and a home address within the study area. The study area was defined as 16 communal sections (the smallest administrative boundary in Haiti) selected by Dr. Jean Pierre to encompass the urban and densely populated parts of Port-au-Prince which would be susceptible to firearm violence. In contrast, use of the official definition for the Port-au-Prince metropolitan area would include a number of less densely populated communal sections encompassing suburban and rural areas. Local expertise suggests less densely populated communities are less susceptible to firearm injuries. Therefore, including less densely populated regions would falsely inflate clustering in urban areas. This is consistent with prior research in the United States, which shows that firearm assaults occur at a higher per capita rate in urban areas [22]. Another advantage of manually selecting urban communal sections for inclusion is that it avoids the exclusion of densely populated areas which do not adhere to formal definitions of the city proper, such as the densely populated informal settlements on the hillsides surrounding the city. The resulting study area is summarized in Fig. 1.

Patients lacking complete address information were excluded. Additionally, there was an implicit exclusion criterion of patients who were not present in the dataset, due to a lack of recording in the logbook or a loss of the logbook page prior to scanning. For the study period (406 days), there were only 273 days with any patient data recorded. Therefore, we can estimate that the records for the study period are 67% complete. Missing data was observed primarily in consecutive periods, ranging from two weeks to four weeks in duration. These periods represent a combination of times when the hospital was closed or when there were missing logbooks. Missing data does not follow any obvious pattern to the research team; however, the frequency of missing data does appear to increase as the study period progresses. It is possible that missing logbook pages will introduce bias—for example extremely high-volume days could be less likely to have a completed logbook. Additionally, the exclusion of missing or incomplete addresses may have preferentially excluded patients with life-threatening injuries, patients from informal settlements, or patients with lower levels of formal education.

### Construction of neighborhoods

**Fig. 2:**
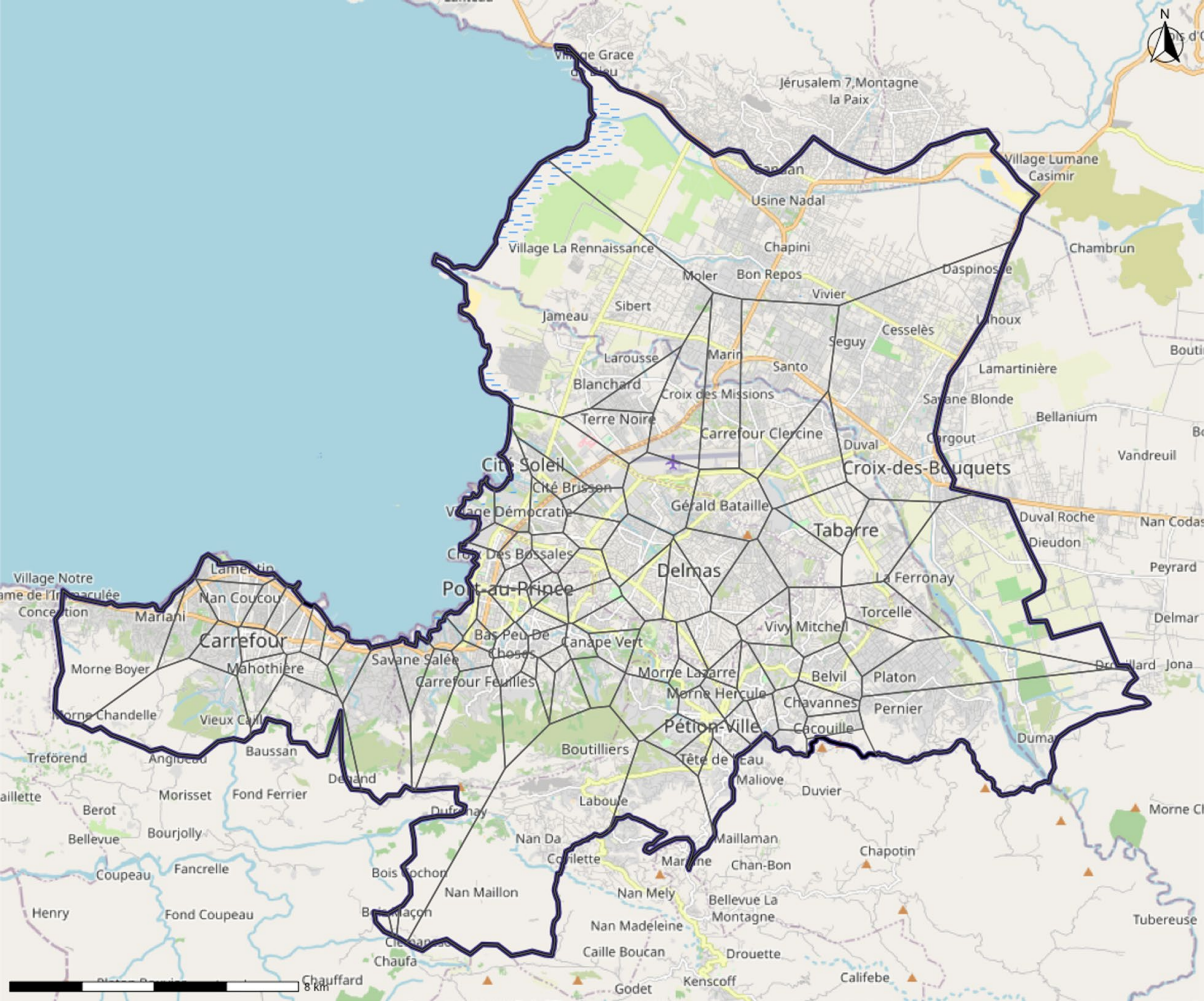
Thiessen polygon estimation of neighborhoods using nodes from OpenStreetMap. [7] Graphic by author

Together, the use of satellite-derived population estimates, and crowdsourced mapping allowed the construction of neighborhood estimations. Neighborhoods were estimated by converting point data representing OpenStreetMap “suburbs” to boundaries using Thiessen polygons (also known as Voronoi diagrams), which were then spatially joined to the boundaries of the study area. This is a reasonable application of Thiessen polygons, as the OpenStreetMap “suburb” point is placed in the center of the neighborhood area [15]. Additionally, Dr. Jean Pierre reviewed the maps to ensure they provided reasonable estimations. This was done by comparing existing maps and local knowledge to the generated neighborhoods, to ensure they provided a realistic representation of boundaries within the city. It was noted that the generated neighborhoods were most accurate in the center of the city, as the use of Thiessen polygons creates greater distortion near the periphery of the study area. This is due to inherent distortion near outer boundaries due to the mathematics underlying the generation of Thiessen polygons, as well as the reduced density of the “suburb” points further from the center of the city. These neighborhoods offered several advantages over existing administrative boundaries. First, they permitted analysis at a higher spatial resolution. Using neighborhood estimates, the included 16 communal sections were able to be converted to 106 neighborhood estimates. The point data obtained from OpenStreetMap was converted to Thiessen polygons in R by using functions in the *sf* package [18]. Specifically, the Thiessen polygons were generated using the *st_voronoi* function, and were split using the *st_cast* function. The resulting polygons were then trimmed to the study area by using the *st_union* function to perform a spatial join. This resolution allowed for operationally relevant results of this study. Secondly, the use of neighborhoods allowed for an increased statistical power, permitting the use of geospatial cluster analysis. Lastly, these-crowdsourced derived neighborhoods may better capture the reality of the divisions within Port-au-Prince. This is especially true for the many informal settlements of the city, which disproportionately face a high burden of firearm injuries, and often cross arbitrary administrative boundaries. The estimated neighborhoods are summarized below in Fig. 2.

### Analysis

Geospatial analysis included both exploratory and cluster analysis. Exploratory analysis included presentation of a kernelled surface, prepared using the R package *ggplot2*’s *stat_density_2d* function [23]. Next, exploratory analysis presented firearm case counts and population adjusted rate for each of the communal sections included in the study area. After this point, all additional analysis was performed at the neighborhood level, which were constructed using Thiessen polygons as previously detailed. The patient dataset contained addresses which had been converted to the spatial resolution of point data as described previously. This was then aggregated to the neighborhood level using a spatial join. The patient dataset used had a temporal resolution of 1 day; however, for purely geospatial analysis, such as Moran’s tests, all cases during the study period were included in the neighborhood case total. Cluster analysis began with a global Moran’s I test for spatial autocorrelation, followed by local Moran’s I test. The global Moran’s I test was performed using the R *spdep* package [24–26]. Local Moran’s I testing was performed with an alpha of 0.10, with three different levels of correction for multiple testing (in order of least to most conservative: unadjusted, performed in the R *spdep* package; manually corrected for a false discovery rate (FDR) [27] corresponding for the 106 neighborhoods, performed in the R *spdep* package and corrected using the base R *stats* package[11]; corrected with a FDR corresponding to the spatial weights matrix, as implemented in the R *rgeoda* package. [28] Three levels of adjustment were performed due to concerns for statistical power, as well as to allow comparison with evolving statistical standards of geospatial analysis. Lastly, cluster analysis was performed in the SaTScan software [29]. While both the local Moran’s I test and SaTScan can be used to describe local patterns and detect hotspots, both were included for two reasons. First, exploratory geospatial analysis was performed in a sequential manner. The local Moran’s I test was performed first, after which the findings of spatial autocorrelation were further explored using the SaTScan analysis. Given the time and computing resources required to perform SaTScan analysis, this was felt to be a worthwhile step. Secondly, and more importantly, the local Moran’s I test and SaTScan provided subtly different insights into the underlying epidemiology—namely, that the local Moran’s I test also detects high-low and low-low spatial autocorrelation, while SaTScan was only used to report positive spatial autocorrelation of cases (which could be comparable to high-high clusters in the local Moran’s I test). Models were run using a discrete Poisson probability model scanning for areas of high rates only [30]. Clusters were limited in size to 50% of the at-risk population. The model was run twice, once using a geospatial analysis (purely spatial) and once using a geospatial-temporal analysis. Time aggregation was performed at the day level, with a limit of temporal clusters to 50% of the study period. Clusters which were purely temporal were not permitted in the SaTScan analysis. SaTScan performed 999 replications, and the threshold for statistically significant clusters was set at < 0.001. All graphics included in this paper were generated by the author by using the R Statistical Software [11]. The following R packages were used in the generation of the figures: *ggplot2* [23], *ggspatial* [31]*,* *sf* [18], *egg* [32], *tmap* [33], *tmaptools* [34], *wesanderson* [35], and *basemaps* [36].

## Results

Of the 8611 patients included in the trauma logbook from HUEH, 342 patients with firearm injuries were identified, representing 4.0% of all emergency department visits. Of this, 269 included patient address information which could be geocoded with sufficient accuracy to be included in the geospatial analysis at a neighborhood level. Of these 269 patients, the geocoded coordinate reported by the Google API was confirmed to have sufficient accuracy for analysis at the neighborhood level in 208 cases. In other words, 61 of the 269 coordinates (23%) were updated during the manual review by Dr. Jean Pierre due to inadequate performance of the API in this setting. Lastly, patients residing outside of the study area were excluded, resulting in 248 cases of firearm injury included in the geospatial analysis.

**Fig. 3:**
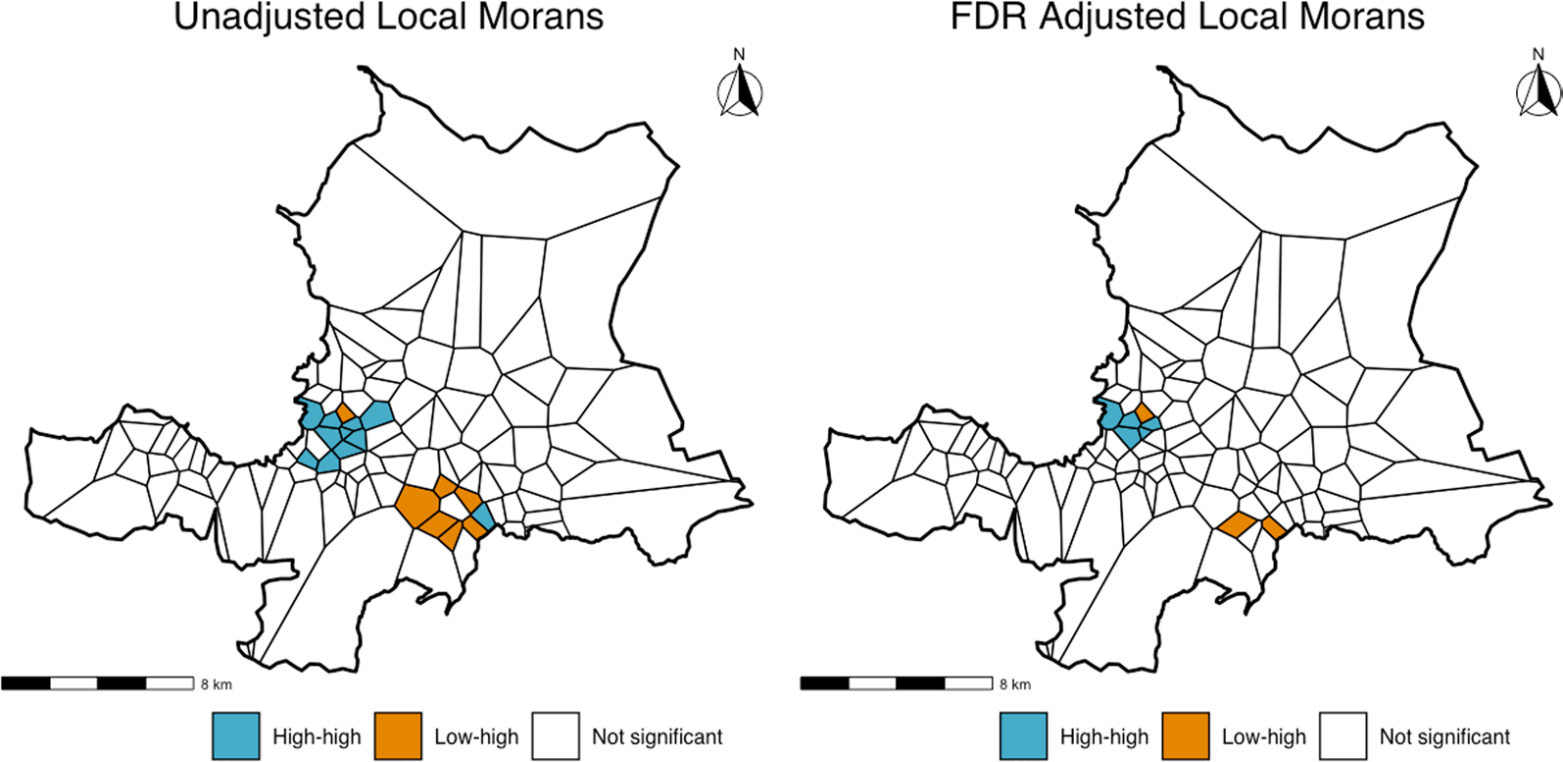
Results from unadjusted and FDR adjusted (for the number of neighborhoods) local Moran’s testing at α = 0.10. Note that more conservative FDR adjustment using the number of neighbor-to-neighbor combinations did not result in any significant clusters. Graphic by author

When interpreting the kernel surface map, it was observed how areas of a high density of firearm injury cases often cross communal section boundaries. This lends support to the use of neighborhoods for further analysis. Additionally, the higher resolution of the kernel surface map demonstrates that firearm injuries appear most prominent in the parts of the city boarding the Caribbean coast. The kernel surface map was not included in this publication in order to protect patient privacy, as it is possible to reverse-engineer point data from the kernelled surface. The exploratory maps generated at the communal section level (excluded for brevity) demonstrated the importance of adjusting for population levels when examining case counts and provided results in a format readily interpretable by policymakers and local stakeholders. Exploratory spatial data analysis continued with Moran’s I testing. Global Moran’s I testing was performed on the population adjusted rate (firearm injuries per 10,000 people) using data aggregated at the neighborhood level and demonstrated significant positive spatial autocorrelation (I = 0.173, p = 0.0003). The results of the local Moran’s testing are shown in Fig. 3 on the following page. which revealed a primary high-high cluster of spatial autocorrelation in the most central parts of PAP near the Caribbean coast. This can be interpreted as neighborhoods with high rates of firearm violence, which border other neighborhoods with high rates of firearm violence. Some significant low–high neighborhoods were also observed, which represent areas with low rates of firearm violence, bordering areas of high rates of firearm violence.

**Fig. 4:**
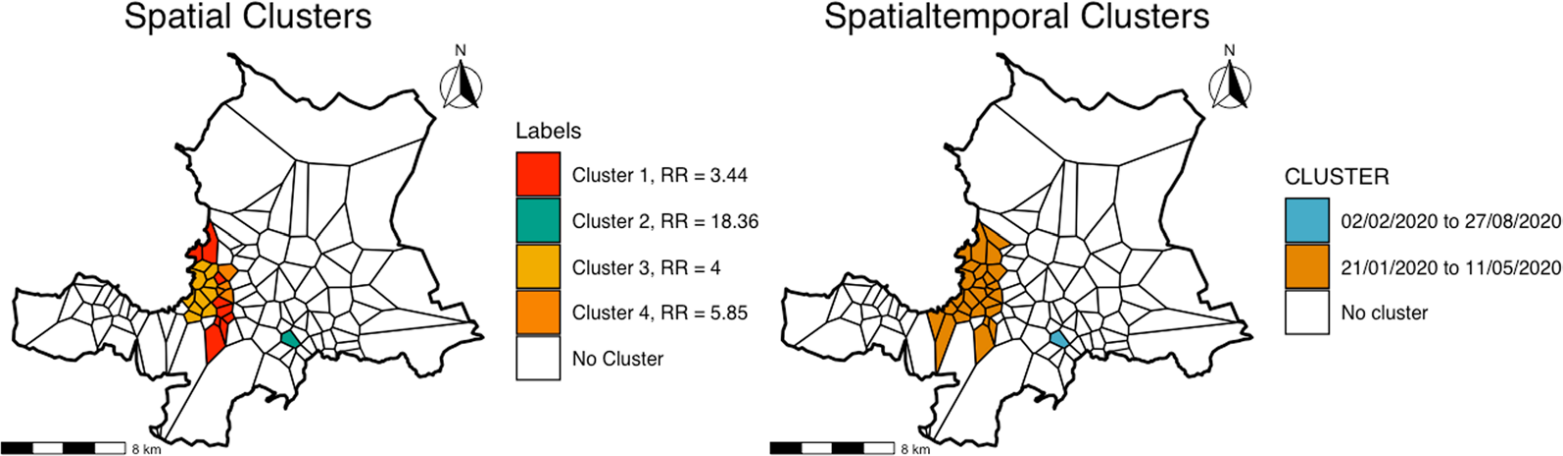
Results from SaTScan analysis of firearm injuries using a Poisson probability model. Labels for spatial clusters include the cluster number, the percentage of total cases captured by the cluster, and the relative risk. Clusters are ordered by cluster number, which is assigned by SaTScan based on the p-value**.** Spatial–temporal clusters are presented with the dates only. Graphic by author

The results of the SaTScan analysis, shown below in Fig. 4, demonstrated both significant spatial and spatial–temporal clusters. The SaTScan output tables are included below as Tables 1 and 2. A single spatial cluster (#1, shown below in red), accounted for 40.7% of all cases of firearm injuries during the study period. It is important to note that this cluster spans several communal sections, again lending support for the need to conduct spatial analysis on a unit smaller than existing administrative boundaries. Additionally, spatial clusters detected by SaTScan suggest that people living in coastal areas extending north and south of the most central portion of the city are also at a higher risk for firearm injuries. Spatial–temporal clusters ranged from spanning several months to a single day. This suggests that the most central parts of the city (the orange spatial–temporal cluster) are faced with chronic violence, while other parts of the city remain susceptible to periods of heightened violence of shorter durations.

**Table 1:** SaTScan spatial analysis output table

Cluster	Neighborhood	Neighborhood Population	Neighborhood Case Count	Neighborhood Relative Risk	Cluster Population	Cluster Case Count	Cluster Relative Risk	P-value
1	Fort National	11,968	6	7.36	593,211	101	3.44	< 0.001
1	Poste Marchand	6317	3	6.90	593,211	101	3.44	< 0.001
1	Bel Air	14,626	6	6.02	593,211	101	3.44	< 0.001
1	Saint Gérard	34,242	13	5.70	593,211	101	3.44	< 0.001
1	Cité de Dieu	22,297	8	5.30	593,211	101	3.44	< 0.001
1	Cité Numéro Un	20,027	7	5.14	593,211	101	3.44	< 0.001
1	Saint Antoine	20,011	6	4.39	593,211	101	3.44	< 0.001
1	Bolosse	41,735	11	3.92	593,211	101	3.44	< 0.001
1	Morne à Tuf	27,762	7	3.70	593,211	101	3.44	< 0.001
1	Bois Verna	22,514	5	3.24	593,211	101	3.44	< 0.001
1	Croix Des Bossales	14,780	3	2.94	593,211	101	3.44	< 0.001
1	Champs de Mars	23,460	4	2.47	593,211	101	3.44	< 0.001
1	La Saline	17,176	2	1.68	593,211	101	3.44	< 0.001
1	Bas Peu De Choses	27,838	3	1.56	593,211	101	3.44	< 0.001
1	Portail Saint Joseph	29,571	3	1.46	593,211	101	3.44	< 0.001
1	Portail Leogane	21,008	2	1.37	593,211	101	3.44	< 0.001
1	Village Démocratie	54,673	5	1.32	593,211	101	3.44	< 0.001
1	Turgeau	28,902	2	0.99	593,211	101	3.44	< 0.001
1	Waaf Jérémie	21,960	1	0.65	593,211	101	3.44	< 0.001
1	Campêche	67,825	3	0.63	593,211	101	3.44	< 0.001
1	Deprez	24,785	1	0.58	593,211	101	3.44	< 0.001
1	Baillergeau	11,358	0	0.00	593,211	101	3.44	< 0.001
1	Saint-Martin	9999	0	0.00	593,211	101	3.44	< 0.001
1	Pacot	18,378	0	0.00	593,211	101	3.44	< 0.001
2	Nerette	16,040	19	18.36	16,040	19	18.36	< 0.001
3	Bel Air	14,626	6	6.02	274,495	62	4.00	< 0.001
3	Saint Gérard	34,242	13	5.70	274,495	62	4.00	< 0.001
3	Cité de Dieu	22,297	8	5.30	274,495	62	4.00	< 0.001
3	Bolosse	41,735	11	3.92	274,495	62	4.00	< 0.001
3	Morne à Tuf	27,762	7	3.70	274,495	62	4.00	< 0.001
3	Croix Des Bossales	14,780	3	2.94	274,495	62	4.00	< 0.001
3	Champs de Mars	23,460	4	2.47	274,495	62	4.00	< 0.001
3	La Saline	17,176	2	1.68	274,495	62	4.00	< 0.001
3	Bas Peu De Choses	27,838	3	1.56	274,495	62	4.00	< 0.001
3	Portail Saint Joseph	29,571	3	1.46	274,495	62	4.00	< 0.001
3	Portail Leogane	21,008	2	1.37	274,495	62	4.00	< 0.001
4	Fort National	11,968	6	7.36	58,323	22	5.85	< 0.001
4	Poste Marchand	6317	3	6.90	58,323	22	5.85	< 0.001
4	Cité Numéro Un	20,027	7	5.14	58,323	22	5.85	< 0.001
4	Saint Antoine	20,011	6	4.39	58,323	22	5.85	< 0.001

**Table 2:** SaTScan spatial–temporal analysis output table

Cluster	Neighborhood	Date range	Neighborhood			Cluster			P-value
			Pop	Case count	RR	Pop	Case count	RR	
1	Cité de Dieu	21/01/2020 to 11/05/2020	22,297	7	17.68	865,530	69	5.67	< 0.001
1	Poste Marchand	21/01/2020 to 11/05/2020	6317	2	17.48	865,530	69	5.67	< 0.001
1	Saint Antoine	21/01/2020 to 11/05/2020	20,011	5	13.96	865,530	69	5.67	< 0.001
1	Cité Numéro Un	21/01/2020 to 11/05/2020	20,027	5	13.94	865,530	69	5.67	< 0.001
1	Fort National	21/01/2020 to 11/05/2020	11,968	3	13.89	865,530	69	5.67	< 0.001
1	Bolosse	21/01/2020 to 11/05/2020	41,735	8	10.82	865,530	69	5.67	< 0.001
1	Morne à Tuf	21/01/2020 to 11/05/2020	27,762	4	8.01	865,530	69	5.67	< 0.001
1	Croix Des Bossales	21/01/2020 to 11/05/2020	14,780	2	7.47	865,530	69	5.67	< 0.001
1	Champs de Mars	21/01/2020 to 11/05/2020	23,460	3	7.08	865,530	69	5.67	< 0.001
1	Saint Gérard	21/01/2020 to 11/05/2020	34,242	4	6.49	865,530	69	5.67	< 0.001
1	Portail Leogane	21/01/2020 to 11/05/2020	21,008	2	5.25	865,530	69	5.67	< 0.001
1	Bois Verna	21/01/2020 to 11/05/2020	22,514	2	4.90	865,530	69	5.67	< 0.001
1	Bel Air	21/01/2020 to 11/05/2020	14,626	1	3.76	865,530	69	5.67	< 0.001
1	Portail Saint Joseph	21/01/2020 to 11/05/2020	29,571	2	3.73	865,530	69	5.67	< 0.001
1	Martissant	21/01/2020 to 11/05/2020	109,011	7	3.59	865,530	69	5.67	< 0.001
1	Savane Salée	21/01/2020 to 11/05/2020	87,372	5	3.18	865,530	69	5.67	< 0.001
1	Deprez	21/01/2020 to 11/05/2020	24,785	1	2.22	865,530	69	5.67	< 0.001
1	Bas Peu De Choses	21/01/2020 to 11/05/2020	27,838	1	1.97	865,530	69	5.67	< 0.001
1	Turgeau	21/01/2020 to 11/05/2020	28,902	1	1.90	865,530	69	5.67	< 0.001
1	Cité L'Eternel	21/01/2020 to 11/05/2020	31,175	1	1.76	865,530	69	5.67	< 0.001
1	Cité Brisson	21/01/2020 to 11/05/2020	35,411	1	1.55	865,530	69	5.67	< 0.001
1	Village Démocratie	21/01/2020 to 11/05/2020	54,673	1	1.00	865,530	69	5.67	< 0.001
1	Campêche	21/01/2020 to 11/05/2020	67,825	1	0.81	865,530	69	5.67	< 0.001
1	Waaf Jérémie	21/01/2020 to 11/05/2020	21,960	0	0.00	865,530	69	5.67	< 0.001
1	La Saline	21/01/2020 to 11/05/2020	17,176	0	0.00	865,530	69	5.67	< 0.001
1	Saint-Martin	21/01/2020 to 11/05/2020	9999	0	0.00	865,530	69	5.67	< 0.001
1	Chancerelles	21/01/2020 to 11/05/2020	27,727	0	0.00	865,530	69	5.67	< 0.001
1	Baillergeau	21/01/2020 to 11/05/2020	11,358	0	0.00	865,530	69	5.67	< 0.001
2	Nerette	02/02/2020 to 27/08/2020	16,040	13	25.19	16,040	13	25.19	< 0.001

## Discussion

First, the results of this study serve as a proof of concept of a novel geospatial methodological approach. Specifically, the combination of local expertise, several open-source datasets, and the use of Thiessen polygons, allowed the generation of a neighborhood level dataset in a setting with limited mapping penetration and significant security concerns which restrict fieldwork. Despite the limitations, this methodology permitted the study of a pressing issue affecting the citizens of Port-au-Prince using geospatial methods. Without the previously mentioned methods, geospatial analysis of firearm injuries in this context may not have been possible. Future researchers may utilize and improve upon these techniques to facilitate geospatial research in areas with similar restrictions. Additionally, this study was able to identify current barriers to geospatial research in Port-au-Prince, and provide specific, realistic suggestions to help overcome these barriers.

Secondly, the results of this analysis demonstrate a high burden of firearm injuries in Port-au-Prince. These firearm injuries are geospatially autocorrelated, clustering in the most central part of the city, and extending outward slightly along the Caribbean coast. The results of the local Moran’s I testing of these areas (shown in Fig. 3) revealed a primary high-high cluster of spatial autocorrelation. This can be interpreted as neighborhoods with high rates of firearm violence, which border other neighborhoods with high rates of firearm violence. In more inland parts of the city, some significant low–high neighborhoods were also observed, which represent areas with low rates of firearm violence, bordering areas of high rates of firearm violence. Local expertise suggests that these are wealthier parts of town with increased security due to a more prominent presence of government forces. The relative risk ratios in Tables 1 and 2 help quantify the variation in the burden of firearm injuries across the city. Many neighborhoods had relative risk ratios greater than five in the geospatial analysis, with relative risk ratios increasing well over ten in the geospatial temporal analysis.

The detection of clustering in space is consistent with the patterns suggested by existing literature of violent firearm injuries, which have shown significant geospatial variation in burden of firearm injuries [37]. The results of the spatial temporal analysis revealed clusters of approximately 4- and 7-months duration, which suggests the burden of firearm injuries in the city is primarily chronic. The presence of temporal clustering may suggest violence due to organized crime, which local expertise and media sources suggest undergoes periodic outbreaks due to conflicts between organized criminal groups. Future research is needed to clarify the forces driving a high burden of firearm injuries in the area study. One hypothesis for why firearm injuries cluster in time is the social contagion model, which states that when someone in a person’s social network becomes a victim of gun violence, that person is at an increased risk for experiencing a firearm injury in the future. Researchers in the U.S. city of Chicago have shown that the majority of firearm injuries in the city could be attributed to social contagion spread [38]. The average time from contagion (exposure to gun violence in one’s social network) to becoming a victim of gun violence in the study by Green et al. was 125 days, which could help explain the longer duration of the temporal clusters detected in the current study [38].

Lastly, when comparing to literature it is important to note the epidemiological drivers of firearm injuries vary between and within nations. For example, one study in the United States and Canada found only a small portion of all firearm injuries were located in geospatial clusters [39]. However, the United States has unique epidemiological drivers of firearm injuries, with a very high burden of self-injuries and accidents (which are especially prominent in rural areas) [39]. If the previously mentioned study were instead limited to assaults with a firearm, the results may have differed. To the authors knowledge, there has been no scientific study of the causal breakdown of firearm injuries in Haiti. Future research could help quantify the relative burdens of interpersonal violence in comparison with other causes, such as unintentional injury and self-harm.

The immediate policy implications of this study must keep in mind the unstable context that Port-au-Prince is currently experiencing. Additionally, a targeted outreach demonstrating lifesaving prehospital bleeding control techniques to areas identified in the clusters has been proposed. After electronic trauma logbooks are implemented, for which funding has been secured, temporal analysis could provide early reporting of firearm injury clusters, so that hospitals can provide additional staff and surgical capacity. As stability and resource availability improves, future implications of this study and these techniques could include staging of prehospital emergency medical services or trial of a violence interruption program. Lastly, understanding the current limitations of geospatial analysis in Port-au-Prince can guide future efforts involving geospatial research in the city.

Specifically, several interventions could facilitate future geospatial research in Port-au-Prince. Participatory community mapping which includes informal settlements could improve the accuracy and resolution of the boundaries between population centers in the city. Future data collection would benefit from the use of a map (either a large physical map or a digital map via a tablet) to record address data, as this would directly capture geospatial coordinates. This is an improvement over relying on coding of addresses, as addresses in some areas of the city may be incomplete or difficult to geocode. For research related to trauma care, future researchers would benefit to additionally record the estimated location at which the injury occurred. A qualitative analysis of patients and stakeholders could help aid in understanding which factors are limiting access to timely trauma care in this specific context. Lastly, researchers could consider a network analysis of existing roadways and hospital distance, which could help capture some of the complexity of transportation routes which patients take to hospitals.

### Limitations

There are significant limitations on the results of this study, and results should be interpreted with caution. The largest bias introduced is using data from only one hospital. It is likely that communities near other hospitals will be systematically excluded from this study. Since HUEH provides care for most public patients in Port-au-Prince, it was determined that the results of this study would be useful for those working to provide trauma care in the city, despite this potential effect on the patient population. Additionally, missing data and small sample size inhibits the detection of temporal clusters. Of note, there were two spatial temporal clusters of a single day duration which were detected during the SaTScan analysis, but which did not meet the threshold for statistical significance. This is important to note for two reasons. First, this finding would support the belief that in addition areas with chronic exposure to firearm injuries, there are also areas which experience episodic exposure to violence due to intermittent conflicts between armed groups–a belief which is evidenced by personal experience of the research team as well as news coverage [3]. Secondly, this illustrates the limitations of power of this study. Other limitations are inherent to the format of data sources used. For example, WorldPop data is a highly interpolated dataset generated from multiple sources, and is not a substitute for current, accurate census data. Another limitation is the use of free text address fields, which are inherently less accurate than coordinates captured directly by a GPS device. Additionally, manual review was only performed by one researcher, future research could use parallel review by multiple independent researchers if manual address review is utilized. Lastly, a final limitation of the study is that the data used in this study predates the current surge of violence in Port-au-Prince, which began in 2021, and should not be assumed to represent the current reality.

## Conclusion

Firearm injuries in Port-au-Prince demonstrate spatial autocorrelation and display epidemiology consistent with violent crime, including geospatial and geospatial-temporal clustering. Despite limitations to geospatial research in this setting, areas most at risk for firearm injuries can be mapped to the neighborhood level. Collaboration between academic institutions, non-state actors, and—most importantly—Haitian medical professionals and other Haitian stakeholders, must continue to mitigate the public health impacts of the epidemic of firearm violence in Port-au-Prince.

## Data Availability

Publicly Available Data: The following datasets analyzed during the current study are available in their corresponding repositories, with citations and web links listed below. 1. Haitian Administrative Boundaries—Humanitarian Data Exchange, Centre National de l’Information Géo-Spatiale (CNIGS). Haiti—Subnational Administrative Boundaries. August 18, 2021. Accessed March 21, 2022. https://data.humdata.org/dataset/cod-ab-hti. 2. WorldPop Population Counts (Constrained Individual Countries 2020, 100 m Resolution, Haiti)—Bondarenko M, Kerr D, Sorichetta A, Tatem A. Census/projection-disaggregated gridded population datasets for 189 countries in 2020 using Built-Settlement Growth Model (BSGM) outputs. University of Southampton. Published online 2020. https://www.worldpop.org/doi/10.5258/SOTON/WP00684. 3. OpenStreetMap—OpenStreetMap Contributors. OpenStreetMap. Published online 2022. Accessed April 18, 2022. http://www.openstreetmap.org. Private Data: Access to the data cited below is restricted due to containing sensitive and identifiable personal health information which would comprise the existing data sharing agreement. 1. Hôpital de l’Université d’Etat d’Haïti (HUEH) Trauma Logbooks—PROTRA Haiti Group. Project Trauma Haiti HUEH Data. November 2019–December 2020. Harvard University. Internal Records.

## References

[1] GBD 2019 Diseases and Injuries Collaborators. Global burden of 369 diseases and injuries in 204 countries and territories, 1990–2019: a systematic analysis for the Global Burden of Disease Study 2019. Lancet. 2020;396:1204–22. 10.1016/S0140-6736(20)30925-9 PMC7567026 33069326

[2] Fene F, Ríos-Blancas MJ, Lachaud J, Razo C, Lamadrid-Figueroa H, Liu M (2020). Life expectancy, death, and disability in Haiti, 1990–2017: a systematic analysis from the Global Burden of Disease Study 2017. Rev Panam Salud Publica.

[3] Guerin O. Haiti: Inside the capital city taken hostage by brutal gangs. BBC News. 2022. https://www.bbc.com/news/world-latin-america-63707429. Accessed 22 Jan 2023.

[4] Pean C. Pattern of Firearm Injuries in a Tertiary Care Center in Port-au-Prince, Haiti. 2022.

[5] Rowhani-Rahbar A, Fan MD, Simonetti JA, Lyons VH, Wang J, Zatzick D (2016). Violence perpetration among patients hospitalized for unintentional and assault-related firearm injury: a case-control study and a cohort study. Ann Intern Med.

[6] Larsen DA, Lane S, Jennings-Bey T, Haygood-El A, Brundage K, Rubinstein RA (2017). Spatio-temporal patterns of gun violence in Syracuse, New York 2009–2015. PLoS ONE.

[7] OpenStreetMap Contributors. OpenStreetMap. 2022. http://www.openstreetmap.org. Accessed 18 Apr 2022.

[8] Institut Haïtien de Statistique et d’Informatique (IHSI). Mars 2015 Population Totale, Population de 18 Ans et Plus Menages et Densites Estimes En 2015. Republique D’Haiti; 2015.

[9] The World Bank. Haiti | Data. 2022. https://data.worldbank.org/country/HT. Accessed 12 Aug 2022.

[10] World Bank. Haiti Overview. 2021. https://www.worldbank.org/en/country/haiti/overview. Accessed 13 May 2022.

[11] R Core Team. R: A Language and Environment for Statistical Computing. Computer software. Vienna, Austria: R Foundation for Statistical Computing; 2022.

[12] Myers SR, Branas CC, Kallan MJ, Wiebe DJ, Nance ML, Carr BG (2011). The use of home location to proxy injury location and implications for regionalized trauma system planning. J Trauma.

[13] Google Developers. Geocoding API. https://developers.google.com/maps/documentation/geocoding/start. Accessed 4 Apr 2022.

[14] Kahle D, Wickham H (2013). ggmap: Spatial Visualization with ggplot2. R J.

[15] OpenStreetMap Contributors. OpenStreetMap Wiki: Tag:place=suburb. 2022. https://wiki.openstreetmap.org/wiki/Tag%3Aplace%3Dsuburb. Accessed 13 May 2022.

[16] Martin Raifer. Overpass Turbo. 2022. https://overpass-turbo.eu. Accessed 13 May 2022.

[17] Padgham M, Lovelace R, Salmon M, Rudis B. osmdata. JOSS. 2017;2:305.

[18] Pebesma E (2018). Simple features for R: standardized support for spatial vector data. R J.

[19] Bondarenko M, Kerr D, Sorichetta A, Tatem A. Census/projection-disaggregated gridded population datasets for 189 countries in 2020 using Built-Settlement Growth Model (BSGM) outputs. University of Southampton. 2020.

[20] Hijmans RJ. raster: Geographic Data Analysis and Modeling. 2023.

[21] Humanitarian Data Exchange, Centre National de l’Information Géo-Spatiale (CNIGS). Haiti—Subnational Administrative Boundaries. 2021. https://data.humdata.org/dataset/cod-ab-hti. Accessed 21 Mar 2022.

[22] Kaufman EJ, Wiebe DJ, Xiong RA, Morrison CN, Seamon MJ, Delgado MK (2021). Epidemiologic trends in fatal and nonfatal firearm injuries in the US, 2009–2017. JAMA Intern Med.

[23] Wickham H (2016). ggplot2: Elegant Graphics for Data Analysis (Use R!).

[24] Bivand RS, Wong DWS (2018). Comparing implementations of global and local indicators of spatial association. TEST.

[25] Bivand RS, Pebesma E, Gomez-Rubio V. Applied spatial data analysis with R, Second edition. Springer, NY; 2013.

[26] Bivand R (2022). R packages for analyzing spatial data: a comparative case study with areal data. Geogr Anal.

[27] Caldas de Castro M, Singer BH (2006). Controlling the false discovery rate: a new application to account for multiple and dependent tests in local statistics of spatial association. Geogr Anal.

[28] Li X, Anselin L. rgeoda: R Library for Spatial Data Analysis. 2021.

[29] Kulldorff M. Information management services, Inc. SaTScanTM v8 0: Software for the spatial and space-time scan statistics. 2009.

[30] Kulldorff M. Bernoulli, discrete Poisson and continuous Poisson models. Commun Stat Theor Methods. 1997;:1481–96.

[31] Dunnington D. ggspatial: Spatial Data Framework for ggplot2. 2021.

[32] Auguie B. egg: Extensions for “ggplot2”: Custom Geom, Custom Themes, Plot Alignment, Labelled Panels, Symmetric Scales, and Fixed Panel Size. 2019.

[33] Tennekes M. tmap: Thematic Maps in *R*. J Stat Softw. 2018;84.

[34] Tennekes M. tmaptools: Thematic Map Tools. 2021.

[35] Ram K, Wickham H. wesanderson: A Wes Anderson Palette Generator. 2018.

[36] Schwalb-Willmann J. basemaps: Accessing Spatial Basemaps in R. 2021.

[37] Dare AJ, Irving H, Guerrero-López CM, Watson LK, Kolpak P, Reynales Shigematsu LM (2019). Geospatial, racial, and educational variation in firearm mortality in the USA, Mexico, Brazil, and Colombia, 1990–2015: a comparative analysis of vital statistics data. Lancet Public Health.

[38] Green B, Horel T, Papachristos AV (2017). Modeling contagion through social networks to explain and predict gunshot violence in Chicago, 2006 to 2014. JAMA Intern Med.

[39] Newgard CD, Sanchez BJ, Bulger EM, Brasel KJ, Byers A, Buick JE (2016). A geospatial analysis of severe firearm injuries compared to other injury mechanisms: event characteristics, location, timing, and outcomes. Acad Emerg Med.

